# QTL Mapping for Grain Zinc and Iron Concentrations in Bread Wheat

**DOI:** 10.3389/fnut.2021.680391

**Published:** 2021-06-09

**Authors:** Yue Wang, Xiaoting Xu, Yuanfeng Hao, Yelun Zhang, Yuping Liu, Zongjun Pu, Yubing Tian, Dengan Xu, Xianchun Xia, Zhonghu He, Yong Zhang

**Affiliations:** ^1^National Wheat Improvement Centre, Institute of Crop Sciences, Chinese Academy of Agricultural Sciences, Beijing, China; ^2^Hebei Laboratory of Crop Genetics and Breeding, Institute of Cereal and Oil Crops, Hebei Academy of Agricultural and Forestry Sciences, Shijiazhuang, China; ^3^Institute of Crop Sciences, Sichuan Academy of Agricultural Sciences, Chengdu, China; ^4^International Maize and Wheat Improvement Center (CIMMYT) China Office, Chinese Academy of Agricultural Sciences, Beijing, China

**Keywords:** *Triticum aestivum*, mineral biofortification, quantitative trait locus, 50K SNP array, KASP marker

## Abstract

Deficiency of micronutrient elements, such as zinc (Zn) and iron (Fe), is called “hidden hunger,” and bio-fortification is the most effective way to overcome the problem. In this study, a high-density Affymetrix 50K single-nucleotide polymorphism (SNP) array was used to map quantitative trait loci (QTL) for grain Zn (GZn) and grain Fe (GFe) concentrations in 254 recombinant inbred lines (RILs) from a cross Jingdong 8/Bainong AK58 in nine environments. There was a wide range of variation in GZn and GFe concentrations among the RILs, with the largest effect contributed by the line × environment interaction, followed by line and environmental effects. The broad sense heritabilities of GZn and GFe were 0.36 ± 0.03 and 0.39 ± 0.03, respectively. Seven QTL for GZn on chromosomes 1DS, 2AS, 3BS, 4DS, 6AS, 6DL, and 7BL accounted for 2.2–25.1% of the phenotypic variances, and four QTL for GFe on chromosomes 3BL, 4DS, 6AS, and 7BL explained 2.3–30.4% of the phenotypic variances. QTL on chromosomes 4DS, 6AS, and 7BL might have pleiotropic effects on both GZn and GFe that were validated on a germplasm panel. Closely linked SNP markers were converted to high-throughput KASP markers, providing valuable tools for selection of improved Zn and Fe bio-fortification in breeding.

## Introduction

Wheat provides the starch, protein, and mineral nutrition needs for 35–40% of the world population (1). Mineral nutrition is crucial for a healthy diet. Over 17% of people suffer from malnutrition worldwide due to lack of mineral nutrition and more than 100,000 children under the age of five die from zinc (Zn) deficiency annually (2–4). The CIMMYT Harvest-Plus program initiated in the early 21st century aimed to address the “hidden hunger” issue by increasing micronutrient concentrations in staple food grains by plant breeding (5). Zn and Fe deficiency were identified as major causes of malnutrition, especially in underdeveloped regions where cereal grains make up most of the food (6).

Zn is a crucial cofactor in many enzymes and regulatory proteins, such as carbonic anhydrase, alkaline phosphatase, and DNA polymerase enzyme synthesis (7). Zn deficiency, first reported in 1961, affects the immune system, taste perception, site, and sexual function (4). Fe deficiency in humans most commonly leads to nutritional anemia in women and children (8). Therefore, it is very important to improve the nutritional quality of wheat by enhancing the Zn (GZn) and Fe (GFe) concentrations in grain (9, 10).

Bio-fortification in wheat breeding demands identification of genetic resources with high GZn and GFe (9). Wide ranges in variation in GZn and GFe have been reported in bread wheat (11–13) and its cultivated and wild relatives (12, 14, 15). Quantitative trait locus (QTL) mapping was used to identify genetic loci affecting GZn and GFe in biparental mapping populations, including recombinant inbred lines (RILs) (16–18). Genome-wide association studies (GWAS) with high-density single-nucleotide polymorphism (SNP) arrays were also used; for example, Alomari et al. (19) performed a GWAS for GZn concentration in 369 European wheats using the 90K and 35K SNP arrays and detected 40 marker–trait associations on chromosomes 2A, 3A, 3B, 4A, 4D, 5A, 5B, 5D, 6D, 7A, 7B, and 7D and 10 candidate genes on chromosomes 3B and 5A. With wide application of molecular markers, such as SSR, DArT, and SNPs, increasing numbers of QTLs for GZn and GFe were detected, including 35 and 32 QTL for GZn and GFe in the A genome, 37 and 30 in the B genome, and 15 and 12 in the D genome, respectively (Supplementary Table 1). The GZn QTL in homoeologous groups 1 to 7 were 9, 10, 13, 11, 13, 12, and 19, respectively, whereas the corresponding numbers of GFe QTL were 6, 17, 10, 8, 15, 7, and 11. QTL pleiotropic for GZn and GFe were identified in homoeologous group 3, 4, 5, and 7 chromosomes.

Cultivar Jingdong 8, with high yield and resistance to stripe rust, leaf rust, and powdery mildew, was released in the early 1990s in the China Northern Winter Wheat Region. It was used widely as a parent in breeding and was verified to have high GZn and GFe levels across environments (13). Bainong AK58, a high yielding cultivar in the Southern Yellow-Huai Valley Winter Wheat Region, has wide adaptability and good resistance to stripe rust, powdery mildew, and lodging, but has lower GZn and GFe. The main goals of the present study were to (1) identify QTL for GZn and GFe in the Jingdong 8/Bainong AK58 RIL population using inclusive composite interval mapping, and (2) develop and validate breeder-friendly markers for marker-assisted selection (MAS) for Zn and Fe biofortification in wheat breeding programs.

## Materials and Methods

### Plant Materials

Two hundred fifty-four F_6_ RILs developed from Jingdong 8/Bainong AK58 cross were used for QTL mapping of GZn and GFe concentrations. A germplasm panel, including 145 cultivars/lines with a wide range of variation in GZn and GFe from the Chinese wheat germplasm bank (13), were used for validation of QTL for GZn and GFe identified in the RIL population.

### Field Trials and Phenotyping

The field trials were conducted at the wheat breeding station of the Institute of Crop Sciences (ICS, CAAS) located at Gaoyi (37°33′N, 114°26′E) and Shijiazhuang (37°27′N, 113°30′E) in Hebei province and Beijing (39°56′N, 116°20′E) during 2016 to 2019 cropping seasons. The parents and RILs were planted in randomized complete blocks with two replications in each environment. Each plot comprised a 1-m row with an inter-row spacing of 20 cm, and a parental check was sown every 30 plots. Standard agronomic practices were applied at each location, along with a soil application of 25 kg/ha ZnSO_4_·7H_2_O in all fields except Beijing.

Grain samples were hand-harvested and cleaned to avoid potential contamination of mineral elements. Micronutrient analysis of grain samples collected from the 2016–2017 cropping season was performed at the Institute of Quality Standards and Testing Technology for Agro-products of CAAS using inductively coupled plasma atomic emission spectrometry (ICP-AES, OPTIMA 3300 DV) after samples were digested in a microwave system with HNO_3_-H_2_O_2_ solution (20). For grain samples from the 2017–2018 and 2018–2019 cropping seasons and the germplasm panel, a “bench-top,” nondestructive, energy-dispersive X-ray fluorescence spectrometry (EDXRF) instrument (model X-Supreme 8000, Oxford Instruments plc, Chengdu) was used to measure GZn and GFe, following the standard method for high-throughput screening of micronutrients in whole wheat grain (21).

### Statistical Analysis

Analysis of variance (ANOVA) was performed by PROC MIXED with method type3 and all effects were treated as fixed in SAS 9.4 software (SAS Institute, Cary, NC). Variance and covariance components for genotype and genotype by environment interaction effects were estimated using PROC MIXED, assuming all effects as random. A similar model was also performed by PROC MIXED with genotype effect as fixed, while environment, replication nested in environment, and interactions involving environment as random, to estimate best linear unbiased estimate (BLUE). Broad-sense heritabilities (*H*_*b*_^2^) on the basis of BLUE value were estimated using the following equation and standard errors were calculated following Holland et al. (22):

$$\begin{array}{l} H_{b}^{2}=\frac{\sigma_{\text{g}}^{2}}{\left(\sigma_{\text{g}}^{2}+\frac{\sigma_{\text{g}\text{e}}^{2}}{\text{e}}+\frac{\sigma_{\varepsilon }^{2}}{\text{r}\text{e}}\right)}\; \end{array}$$

where $\sigma_{g}^{2}$ represents the variance of genotypes, $\sigma_{ge}^{2}$ and $\sigma_{\varepsilon }^{2}$represent the variances of genotype × environment interaction and error, and *e* and *r* represent environments and number of replicates per environment, respectively. Phenotypic and genotypic correlations and their standard errors were estimated after Becker (23). Student's *t* test was performed by PROC TTEST.

### SNP Genotyping and QTL Analysis

Genomic DNA extracted from fresh seedling leaves of RILs and parents by CTAB method (24) were used for genotyping by the wheat 50K SNP Array. The wheat 50K SNP Array was developed in collaboration by CAAS and Capital-Bio, Beijing, China (https://www.capitalbiotech.com/). Linkage analysis was performed with JoinMap v4.0 using the regression mapping algorithm (25). QTL analysis was performed by inclusive composite interval mapping with the ICIM-ADD function using QTL IciMapping v4.1 (http://www.isbreeding.net). Phenotypic values of RILs averaged from two replicates in each environment and BLUE value across nine environments were used for analyses. QTL detection was done using a logarithm of odds (LOD) threshold of 2.5. Pleiotropic QTL were analyzed using the module JZmapqtl of multi-trait composite interval mapping (MCIM) in Windows QTL Cartographer v2.5 (26). QTL pyramids were plotted using ggplot2 in R (27). Physical maps for the positional comparisons of GZn and GFe QTL with previous reports were exhibited using MapChart v2.3 (28).

### Conversion of SNPs to KASP Markers

Kompetitive Allele Specific PCR (KASP) markers were developed from SNPs tightly linked with the targeted QTL, each including two competitive allele-specific forward primers and one common reverse primer. Each forward primer incorporated an additional tail sequence that corresponds to only one of the two universal fluorescence resonance energy transfers. Primers were designed from information in the PolyMarker website (http://polymaker.tgac.ac.uk/). PCR procedures and conditions followed Chandra et al. (29). Gel-free fluorescence signal scanning and allele separation were conducted by microplate reader (Multiscan Spectrum BioTek, Synegy/H1) with Klustercaller 2.24.0.11 software (LGC, Hoddesdon, UK) (30).

## Results

### Phenotypic Evaluation

**Figure 1:**
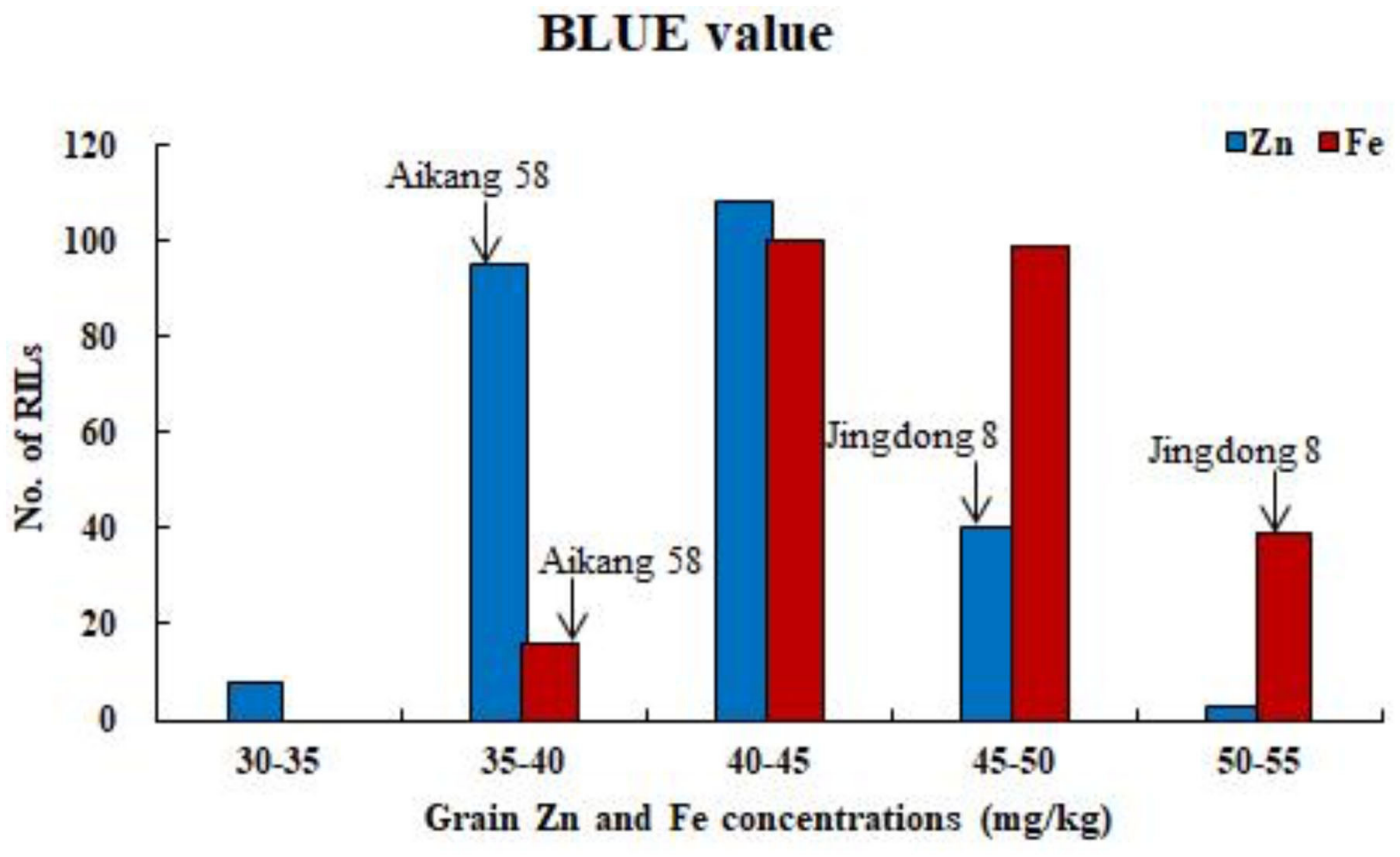
Frequency distributions of GZn and GFe based on BLUE value across nine environments for 254 RILs in the Jingdong 8/Bainong AK58 population.

**Figure 2:**
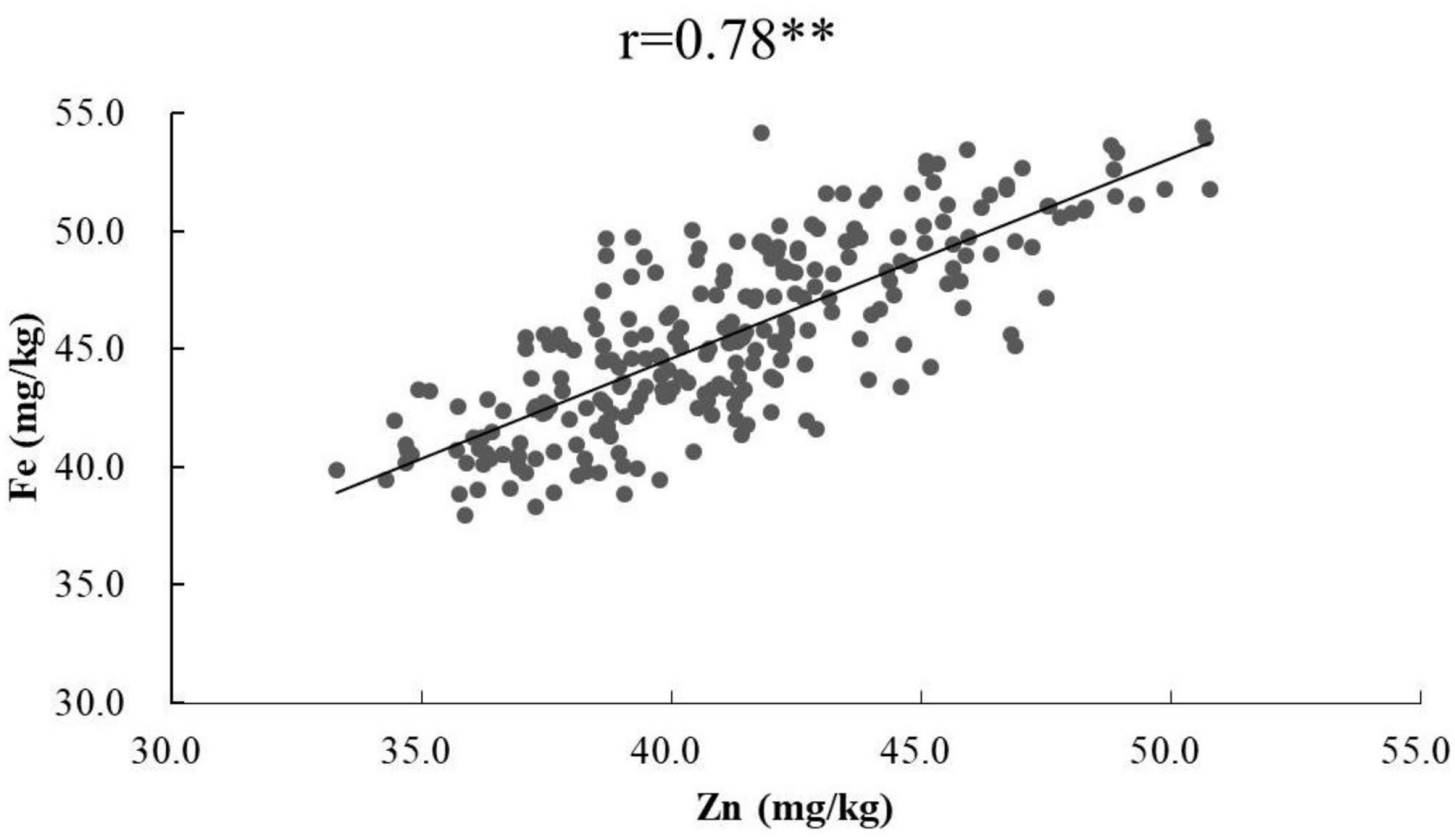
Phenotypic correlation of GZn with GFe based on BLUEs across nine environments in the Jingdong 8/Bainong AK58 RIL population.

ANOVA showed that GZn and GFe were significantly influenced by lines, environments, and line by environment interaction effects, with line by environment interaction effects contributing the highest variation, followed by line and environment effects (Table 1). The broad-sense heritabilities of GZn and GFe were 0.36 ± 0.03 and 0.39 ± 0.03, respectively. Jingdong 8 accumulated significantly higher GZn and GFe than Bainong AK58. Wide-ranging continuous variation among the RILs suggests polygenic inheritance (Table 2, Figure 1). Significant and positive correlations of GZn (*r* = 0.25–0.67, *P* < 0.01) and GFe (*r* = 0.26–0.70, *P* < 0.01) were observed across the nine environments (Table 3). Additionally, positive phenotypic and genotypic correlations between GZn and GFe (*r* = 0.78 ± 0.01 and 0.81 ± 0.03, *P* < 0.001) (Figure 2), indicated that GZn and GFe were, to some degree, simultaneously accumulated.

**Table 1:** Analysis of variance of GZn and GFe in 254 RILs derived from the cross Jingdong 8/Bainong AK58 grown in nine environments.

**Source of variation**	**DF**	**Sum square**	
		**Zn**	**Fe**
Line	253	39,148^**^	50,429^**^
Environment (Env)	8	67,264^**^	24,847^**^
Line × Env	2,024	74,960^**^	91,715^**^
Rep (Env)	9	3,385^**^	1,658^**^
Error	2021	46,076	49,910
Heritability		0.36 ± 0.03	0.39 ± 0.03

** *Significant at P < 0.01*.

**Table 2:** Mean and range of GZn and GFe (mg/kg) in the Jingdong 8/Bainong AK58 RIL population among nine environments.

		**Parents**		**RILs**	
**Trait**	**Environment**	**Jingdong 8**	**Bainong AK58**	**Range**	**Mean ± SD**
Zn (mg/kg)	E1	42.1	35.1	25.4–52.6	38.9 ± 4.6
	E2	41.5	34.1	25.2–56.6	39.1 ± 5.6
	E3	53.4	46.4	29.5–60.7	43.5 ± 5.7
	E4	41.3	34.5	28.7–52.6	38.0 ± 4.1
	E5	52.1	44.1	33.5–62.2	46.4 ± 4.7
	E6	46.2	36.9	28.9–54.3	41.3 ± 5.3
	E7	40.7	33.7	25.7–49.0	34.6 ± 3.9
	E8	55.0	42.3	34.6–62.5	47.6 ± 6.0
	E9	48.6	34.7	27.0–57.9	40.0 ± 5.8
Fe (mg/kg)	E1	51.0	43.0	32.8–62.6	47.3 ± 5.2
	E2	53.5	40.9	34.5–68.9	48.0 ± 6.5
	E3	53.2	42.8	34.2–64.0	48.5 ± 6.3
	E4	46.6	40.2	35.3–52.3	42.2 ± 3.2
	E5	49.8	42.3	37.0–59.5	44.9 ± 3.8
	E6	49.2	34.5	31.1–65.1	42.7 ± 5.5
	E7	54.2	37.8	32.0–67.2	45.0 ± 6.7
	E8	56.6	39.7	33.9–69.2	49.2 ± 6.2
	E9	55.0	38.0	28.0–63.9	47.1 ± 6.4

*E1–E9, Shijiazhuang 2016–2017, Gaoyi 2016–2017, Beijing 2016–2017, Shijiazhuang 2017–2018, Gaoyi 2017–2018, Beijing 2017–2018, Shijiazhuang 2018–2019, Gaoyi 2018–2019, and Beijing 2018–2019*.

**Table 3:** Pearson correlation coefficients of GZn and GFe in the Jingdong 8/Bainong AK58 RIL population among nine environments.

**Environment**	**E1**	**E2**	**E3**	**E4**	**E5**	**E6**	**E7**	**E8**	**E9**
E1		0.45^***^	0.32^***^	0.40^***^	0.37^***^	0.48^***^	0.36^***^	0.47^***^	0.48^***^
E2	0.70^***^		0.25^***^	0.41^***^	0.46^***^	0.52^***^	0.27^***^	0.43^***^	0.39^***^
E3	0.52^***^	0.52^***^		0.40^***^	0.33^***^	0.40^***^	0.34^***^	0.34^***^	0.36^***^
E4	0.44^***^	0.47^***^	0.47^***^		0.49^***^	0.57^***^	0.40^***^	0.52^***^	0.51^***^
E5	0.40^***^	0.46^***^	0.42^***^	0.40^***^		0.50^***^	0.31^***^	0.58^***^	0.46^***^
E6	0.51^***^	0.51^***^	0.44^***^	0.53^***^	0.51^***^		0.50^***^	0.67^***^	0.60^***^
E7	0.53^***^	0.54^***^	0.46^***^	0.55^***^	0.51^***^	0.63^***^		0.47^***^	0.43^***^
E8	0.41^***^	0.47^***^	0.45^***^	0.51^***^	0.48^***^	0.53^***^	0.62^***^		0.55^***^
E9	0.26^***^	0.28^***^	0.31^***^	0.38^***^	0.32^***^	0.38^***^	0.41^***^	0.39^***^	

*** *Significant at P < 0.001.*

*Upper right triangle: Correlation coefficients between environments for GZn.*

*Lower left triangle: Correlation coefficients between environments for GFe.*

*E1–E9, Shijiazhuang 2016–2017, Gaoyi 2016–2017, Beijing 2016–2017, Shijiazhuang 2017–2018, Gaoyi 2017–2018, Beijing 2017–2018, Shijiazhuang 2018–2019, Gaoyi 2018–2019, and Beijing 2018–2019*.

### Linkage Map Construction

Among 54,680 SNP markers in the 50K SNP array, 20,060 were polymorphic after removal of markers that were monomorphic, absent in more than 20% of assays, and minor allele frequency was <30%. A high-density linkage map spanning 3423 cM and including all 21 chromosomes was constructed using 3328 representative SNP markers of each bin. The average chromosome length was 163 cM, ranging from 116.72 cM (1B) to 237.40 cM (5A) (Supplementary Table 2).

### QTL Mapping of GZn and GFe using ICIM and MCIM

**Figure 3:**
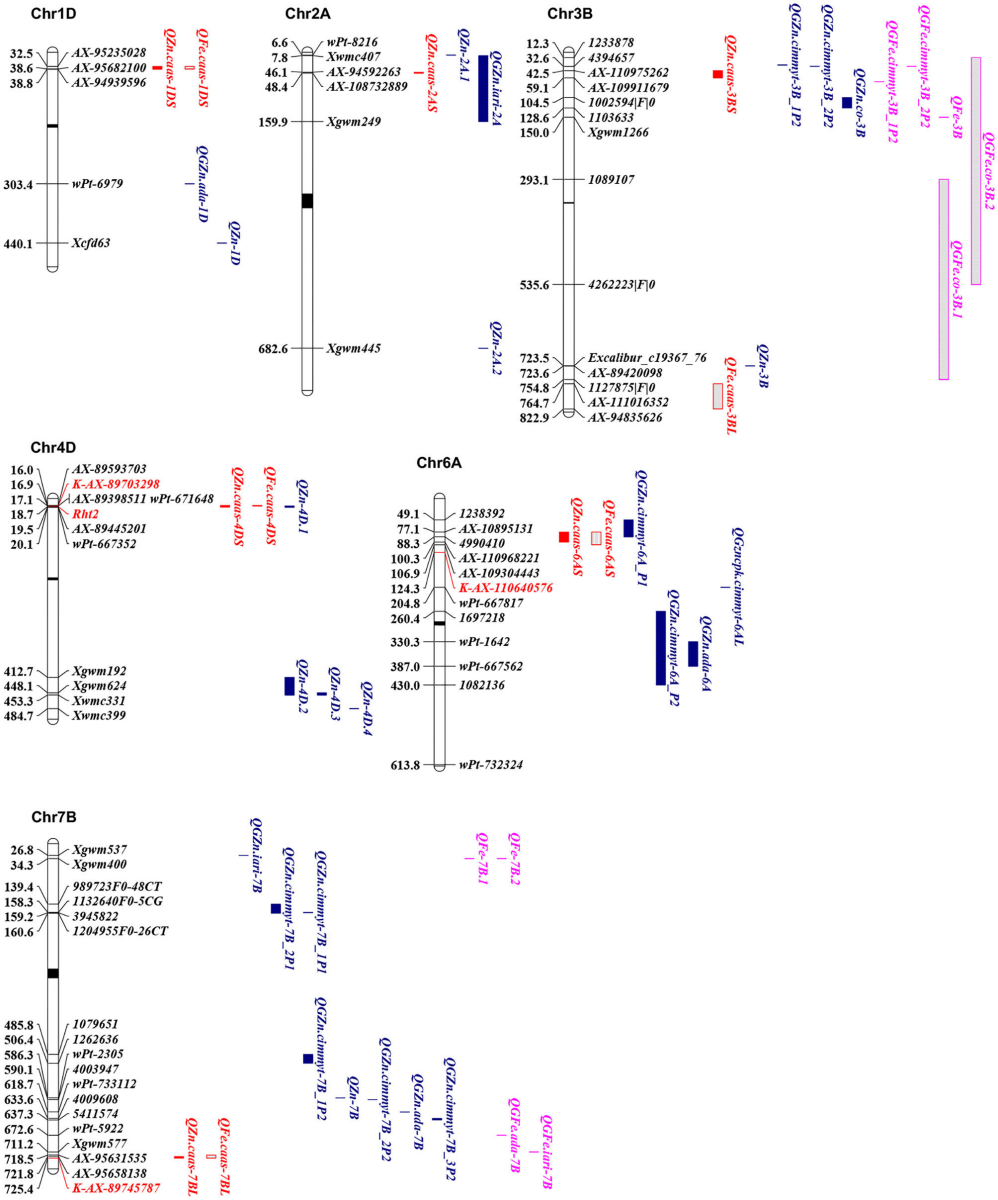
Physical maps for the positional comparisons of GZn and GFe QTL reported on chromosomes 1DS, 2AS, 3BL, 4DS, 6AS, and 7BL with those identified in the present study. QTL linked markers are shown on the right, physical positions are shown on the left, and centromere is shown in the middle (black bar). KASP markers developed in the present study were shown in red. QTL for GZn and GFe in the present study were in red; QTL for GZn in previous studies were in blue; QTL for GFe in previous studies were in purple.

Seven QTL for GZn were mapped on chromosomes 1DS, 2AS, 3BS, 4DS, 6AS, 6DL, and 7BL, explaining 2.2–25.1% of the phenotypic variances (Table 4, Supplementary Table 3, and Figure 3), with five favorable alleles coming from Jingdong 8, and with the other two, i.e., *QZn.caas-1DS* and *QZn.caas-3BS*, coming from Bainong AK58. Four QTL for GFe were detected on chromosomes 3BL, 4DS, 6AS, and 7BL, explaining 2.3–30.4% of the phenotypic variances (Table 4, Supplemenatry Table 3, and Figure 3), with all superior alleles coming from Jingdong 8. Among these QTL, three were identified for both GZn and GFe at the same or overlapping location on chromosomes 4DS, 6AS, and 7BL.

**Table 4:** QTL for GZn and GFe identified by inclusive composite interval mapping in the Jingdong 8/Bainong AK58 RIL population.

**Trait**	**QTL**	**Environment**	**Physical interval^**a**^**	**Marker interval**	**LOD^**b**^**	**PVE(%)^**c**^**	**Add^**d**^**
Zn	*QZn.caas-1DS*	E3	32.5–38.8	*AX-95235028–AX-94939596*	3.0	3.5	1.1
		E6			2.7	3.4	0.9
		E8			6.0	6.0	1.5
	*QZn.caas-2AS*	E5	46.1–48.4	*AX-94592263–AX-108732889*	4.1	2.2	−1.1
		E8			9.2	9.3	−1.8
	*QZn.caas-3BS*	E2	42.5–59.1	*AX-110975262–AX-109911679*	3.7	5.7	1.3
		E4			4.8	5.5	1.0
	*QZn.caas-4DS*	E1	16.0–19.5	*AX-89593703–AX-89445201*	10.8	12.1	−1.8
		E2			4.6	7.2	−1.5
		E4			9.0	10.7	−1.4
		E5			4.4	2.4	−1.1
		E6			17.1	25.1	−2.5
		E7			3.5	4.9	−0.9
		E8			13.1	14.3	−2.3
		E9			8.5	11.2	−1.9
	*QZn.caas-6AS*	E4	77.1–100.3	*AX-108951317–AX-110968221*	4.4	5.2	−1.0
		E6			3.7	4.8	−1.1
	*QZn.caas-6DL*	E3	454.1–459.4	*AX-109058428–AX-111841126*	7.3	8.5	−1.7
		E4			3.0	3.5	−0.8
	*QZn.caas-7BL*	E1	721.8–725.4	*AX-95658138–AX-89745787*	4.2	4.3	−1.0
		E3			5.4	6.4	−1.5
		E6			5.4	6.9	−1.3
		E8			6.3	6.3	−1.5
		E9			5.0	6.2	−1.4
Fe	*QFe.caas-3BL*	E5	764.7–822.9	*AX-111016352–AX-94835626*	3.1	5.8	−0.9
		E6			2.8	2.9	−0.9
	*QFe.caas-4DS*	E1	16.0–17.1	*AX-89593703–AX-89398511*	16.8	20.4	−2.5
		E2			18.7	27.0	−3.4
		E3			12.3	19.4	−2.7
		E4			24.1	24.3	−1.9
		E5			6.2	9.0	−1.1
		E6			20.6	25.6	−2.7
		E7			20.8	30.4	−3.4
		E8			11.2	5.5	−2.3
		E9			4.6	2.3	−1.7
	*QFe.caas-6AS*	E1	77.1–106.9	*AX-108951317–AX-109304443*	4.7	5.1	−1.2
		E6			7.0	7.5	−1.5
	*QFe.caas-7BL*	E1	718.5–725.4	*AX-95631535–AX-89745787*	2.8	2.9	−0.9
		E6			6.2	6.9	−1.4

a *Physical interval; Mb, according to IWGSC RefSeq v1.0 (31), http://www.wheatgenome.org/.*

b *LOD; likelihood of odds ratio for genetic effects.*

c *PVE; percentage of phenotypic variance explained by individual QTL.*

d *Add; Additive effect of QTL; negative values indicate that the superior allele came from Jingdong 8, whereas positive values indicate that the superior allele was from Bainong AK58.*

*E1–E9, Shijiazhuang 2016–2017, Gaoyi 2016–2017, Beijing 2016–2017, Shijiazhuang 2017–2018, Gaoyi 2017–2018, Beijing 2017–2018, Shijiazhuang 2018–2019, Gaoyi 2018–2019, and Beijing 2018–2019*.

Three chromosomal intervals were detected using MCIM including 4DS, 6AS, and 7BL, corresponding to co-localized QTL for GZn and GFe by ICIM-ADD (Table 5). Two intervals on chromosomes 4DS and 6AS were detected in most environments for GZn and GFe, while the one on chromosome 7BL was found in most environments for GZn but only one environment for GFe.

**Table 5:** Chromosomal intervals for GZn and GFe identified by multi-trait composite interval mapping (MCIM).

**Chromosomes**	**Flanking markers**	**Physical position (Mb)**	**Traits (Environment)**
4DS	*AX-89593703*–*AX-89398511*	16.0–17.1	GZn (E1, E2, E4, E6, E7, E8, E9, BLUE value)
			GFe (E1, E2, E4, E5, E6, E7, E8, E9, BLUE value)
6AS	*AX-108951317–AX-110968221*	77.1–100.3	GZn (E1, E2, E4, E6, E7, BLUE value)
			GFe (E1, E2, E4, E6, BLUE value)
7BL	*AX-95658138–AX-89745787*	721.8–725.4	GZn (E1, E3, E6, E7, E8, E9, BLUE value)
			GFe (E6)

### QTL Pyramids and Validation

It indicated that superior alleles of pleiotropic QTL on 4DS, 6AS, and 7BL were all from Jingdong 8. Accumulation effect of the three co-localization QTL for GZn and GFe was calculated based on the closely linked markers. The average concentration of GZn increased from 37.79 to 44.43 mg/kg and that of GFe increased from 41.02 to 50.37 mg/kg, with lines containing zero to three favorable alleles (Supplementary Figure 1).

Flanking SNPs closely linked to the QTL on chromosomes 4DS and 7BL and a SNP near QTL region of 6AS were converted to KASP markers and validated in the germplasm panel (Tables 6, 7). Cultivars with the same superior allele as Jingdong 8 had significantly higher GZn and GFe than those with the inferior allele from Bainong AK58 for all QTL, except for *QFe.caas-6AS*. The difference between the superior and inferior allele of the QTL on chromosomes 4DS, 6AS, and 7BL was 1.7, 2.8, and 3.5 mg/kg for GZn and 1.4, 1.0, and 4.7 mg/kg for GFe, respectively (Table 7).

**Table 6:** Kompetitive allele specific PCR (KASP) markers converted from single-nucleotide polymorphisms (SNPs) tightly linked to identified QTL on three chromosomes.

**Chromosome**	**SNP name**	**Physical position (Mb)**	**KASP primer**	**Primer sequence**
4DS	*AX-89703298*	16.9	*K-AX-89703298*	5′-GAAGGTGACCAAGTTCATGCTCTAACCATTGGATAGGGCGAC-3′
				5′-GAAGGTCGGAGTCAACGGATTCTAACCATTGGATAGGGCGAA-3′
				5′-CCCAGCTTCAGCCCATGA-3′
6AS	*AX-110640576*	124.3	*K-AX-110640576*	5′-GAAGGTGACCAAGTTCATGCTCACAGATGTTCTCCACTCTCTG-3′
				5′-GAAGGTCGGAGTCAACGGATTCACAGATGTTCTCCACTCTCTC-3′
				5′-CCCTCCAAGGTCCATGGGT−3′
7BL	*AX-89745787*	725.4	*K-AX-89745787*	5′-GAAGGTGACCAAGTTCATGCTGGAGGACATTGTGCAACCG-3′
				5′-GAAGGTCGGAGTCAACGGATTGGAGGACATTGTGCAACCT-3′
				5′-AGGATTGGTTCTGCAATCCA-3′

**Table 7:** Mean values of GZn and GFe for genotype classes in the germplasm panel.

**Trait**	**QTL**	**Marker**	**Genotype**	**Number**	**Mean (mg/kg)**	***T* value**
GZn	*QZn.caas-4DS*	*K-AX-89703298*	CC	79	32.4	−2.28^*^
			AA	66	30.7	
	*QZn.caas-6AS*	*K-AX-110640576*	GG	19	34.0	−2.54^*^
			CC	126	31.2	
	*QZn.caas-7BL*	*K-AX-89745787*	GG	11	34.7	−2.41^*^
			TT	134	31.4	
GFe	*QFe.caas-4DS*	*K-AX-89703298*	CC	79	39.4	−2.58^*^
			AA	66	38.0	
	*QFe.caas-6AS*	*K-AX-110640576*	GG	19	39.6	−1.18
			CC	126	38.6	
	*QFe.caas-7BL*	*K-AX-89745787*	GG	11	43.1	−2.55^*^
			TT	134	38.4	

* *Significant at P < 0.05*.

## Discussion

### Comparisons With Previous Reports

In this study, QTL for GZn and GFe were mapped on chromosomes 1D, 2A, 3B, 4D, 6A, 6D, and 7B, and on chromosomes 3B, 4D, 6A, and 7B, respectively. Previously identified QTL are summarized in Supplementary Table 1 and partly shown in Figure 3. In addition to consensus maps, the IWGSC RefSeq v1.0 Chinese Spring reference sequence (31) was used for comparisons of QTL identified in different studies.

#### QZn.caas-1DS

*QZn.caas-1DS*, flanked by SNP markers *AX-95235028* and *AX-94939596* at 32.5–38.8 Mb, was detected in three environments. Velu et al. (18) identified *QGZn.ada-1D* linked with a DArT marker *wPt-6979* at 303.4 Mb. Gorafi et al. (32) detected a QTL linked with SSR marker *Xcfd63* at physical position 440 Mb. The present QTL appears to be new.

#### QZn.caas-2AS

*QZn.caas-2AS*, flanked by *AX-94592263* and *AX-108732889* at physical positions of 46.1 and 48.4 Mb, was identified in two environments. Peleg et al. (33) identified *QZn-2A.1* and *QZn-2A.2* linked with *wPt-8216* and *Xgwm445* at 6.6 and 682.6 Mb, respectively. Krishnappa et al. (34) mapped *QGZn.iari-2A* flanking by *Xwmc407* and *Xgwm249* at physical position 28.2 and 159.9 Mb, respectively. *QZn.caas-2AS* detected in the present study was located within the region of *QGZn.iari-2A*; therefore, these two QTL may be the same.

#### QZn.caas-3BS

*QZn.caas-3BS*, flanked by *AX-110975262* and *AX-109911679* at physical positions of 42.5 and 59.1 Mb, was detected in two environments. Crespo-Herrera et al. (17) identified two QTL for GZn on this chromosome. *QGZn.cimmyt-3B_2P2* was at the physical position 32.6 Mb linked with DArT markers *4394657*, and *QGZn.cimmyt-3B_1P2* flanked by 3533713 and 1007339 is much more near the distal end of 3BS than *QGZn.cimmyt-3B_2P2* on the basis of the genetic map, although both markers were not on chromosome 3B with the result of blast. Furthermore, Liu et al. (35) mapped *QGZn.co-3B* flanked by DArT markers *1002594|F|0* and *1103633* at physical positions of 104.5 and 128.6 Mb, respectively. Alomari et al. (19) identified a locus for GZn on chromosome 3BL, linked with *AX-89420098* at 723.5 Mb. Thus, the previous QTL were around 10 Mb from *QZn.caas-3BS*, indicating that *QZn.caas-3BS* is likely a new QTL.

#### QFe.caas-3BL

*QFe.caas-3BL*, flanked by *AX-111016352* and *AX-94835626* at physical positions of 764.7 and 822.9 Mb, was detected in two environments. Crespo-Herrera et al. (17) identified two QTL for GFe that were at the similar position as QTL for GZn as mentioned previously, both of which were on the short arm of chromosome 3B. Peleg et al. (33) mapped a QTL on chromosome 3B, closely linked with *Xgwm1266* at physical position 150 Mb. Liu et al. (35) identified *QGFe.co-3B.1* and *QGFe.co-3B.2* flanked by DArT markers *1089107* and *1127875|F|0, 1233878-4262223|F|0* at physical positions 37.2–754.8 and 12.3–536.6 Mb, respectively. These five QTL were at least 10 Mb distant from *QFe.caas-3BL*. Therefore, *QFe.caas-3BL* is likely a new QTL for GFe.

#### *QZn.caas-4DS* and *QFe.caas-4DS*

*QZn.caas-4DS* and *QFe.caas-4DS*, flanked by *AX-89593703* and *AX-89445201* at physical positions of 16.0 and 19.5 Mb were detected in eight and nine environments, respectively. Pu et al. (36) identified a QTL for GZn at the same position, flanked by *wPt-671648* and *wPt-667352* located between 17.1 and 20.1 Mb on chromosome 4D, with reduced height gene *Rht2* (*Rht-D1b*) located in this region. Using a limited number of isogenic lines, Graham et al. (37) found that lower GZn and GFe in wheat was associated with reduced height genes. Velu et al. (38) verified this association using nine bread wheat (*Triticum aestivum*) and six durum (*T. turgidum*) isogenic line pairs differing at the *Rht1* (*Rht-B1*) locus and one bread wheat pair differing at the *Rht2* locus, indicating that the presence of reduced height genes decreased GZn by 1.9 to 10.0 ppm and GFe by 1.0 to 14.4 ppm. In this study, Bainong AK58 carried *Rht2* (*Rht-D1b*), while Jingdong 8 had *rht2* (*Rht-D1a*) (39). A gene-specific KASP marker *K-AX-86170701* was identified for *Rht2* (40), and lines with allele from Bainong AK58 had significantly lower GZn and GFe than that with allele from Jingdong 8 (Supplementary Figure 2). Therefore, it was possible that the lower concentrations of Zn and Fe in Bainong AK58 was associated with the *Rht2* allele.

#### *QZn.caas-6AS* and *QFe.caas-6AS*

*QZn.caas-6AS* and *QFe.caas-6AS*, flanked by *AX-108951317* and *AX-110968221* at physical positions of 77.1 and 106.9 Mb, were detected in four environments. No QTL for GFe was detected on chromosome 6AS previously, while two QTL for GZn were reported. Crespo-Herrera et al. (17) identified *QGZn.cimmyt-6A_P1*, linked with *1238392* and *4990410* at physical positions of 49.1 and 88.2 Mb. Hao et al. (16) mapped *QGZn.cimmyt-6AL* at 204.8 Mb with nearest marker *wPt-667817*. The present QTL was somewhat near the *QGZn.cimmyt-6A_P1*, indicating that they might be the same.

#### QZn.caas-6DL

*QZn.caas-6DL*, flanked by *AX-109058428* and *AX-11184112* at physical positions of 454.1 and 459.4 Mb, was detected in two environments. It is likely a new one since no previous QTL for GZn was mapped on this chromosome.

#### *QZn.caas-7BL* and *QFe.caas-7BL*

Markers *AX-95631535* and *AX-89745787* at positions 718.5 and 725.4 Mb flanking *QZn.caas-7BL* and *QFe.caas-7BL* are distally located on chromosome 7BL. Several QTL were previously identified on this chromosome. Krishnappa et al. (34) mapped *QGZn.iari-7B* with closest marker *Xgwm537* at 26.8 Mb. Peleg et al. (33) detected *QZn-7B* linked with *wPt-2305* at 586.3 Mb. Crespo-Herrera et al. (17) identified five QTL, including *QGZn.cimmyt-7B_2P1, QGZn.cimmyt-7B_1P1, QGZn.cimmyt-7B_1P2, QGZn.cimmyt-7B_2P2*, and *QGZn.cimmyt-7B_3P2* at physical positions of 139.4–160.6, 158.3–159.2, 485.8–506.4, 590.1, and 633.6–637.3 Mb, respectively. Velu et al. (18) reported *QGZn.ada-7B*, which was located at around 618.7 Mb with closely linked marker *wPt-733112*. All these eight QTL were well proximal (>80 Mb) from the QTL in this study, indicating that *QZn.caas-7BL* was reported for the first time. In addition, four QTL for GFe were mapped on chromosome 7B, among which two of them were at the same physical position of 34.3 Mb (*QFe-7B.1* and *QFe-7B.2*), and the other two were at 672.6 Mb (*QGFe.ada-7B*) and 711.2 Mb (*QGFe.iari-7B*), respectively (18, 28, 29, 41). *QGFe.iari-7B* and *QFe.caas-7BL* might be the same, since their distance is <10 Mb.

### Pleiotropic Effects of QTL

The co-localization QTL for GZn and GFe on chromosomes 4DS, 6AS, and 7BL might be pleiotropic QTL based on the same or overlapping region detected using MCIM, in agreement with the significant positive phenotypic and genotypic correlations (*r* = 0.78 ± 0.01 and 0.81 ± 0.03, *P* <0.01) between GZn and GFe. Gorafi et al. (32) identified a significant and positive phenotypic correlation between GZn and GFe (*r* = 0.78) and a pleiotropic QTL on chromosome 5D; significant and positive correlations between GZn and GFe were also found in other studies (11, 42). It has been reported that some transporters, chelators, and genes regulated GZn and GFe simultaneously in a high frequency (10, 43). These findings indicated that Zn and Fe could be improved simultaneously in breeding programs targeting mineral biofortification.

### Potential Implication in Wheat Breeding

MAS has been applied in crop breeding for more than two decades. Therefore, it would be effective for traits that were controlled by low numbers of major QTL (37). The phenotypic analysis on GZn and GFe was time-consuming and laborious, indicating that identification of molecular markers linked to GZn and GFe would be of interest for improvement of nutritional quality in wheat. In the present study, pleiotropic QTL on chromosomes 4D, 6A, and 7B were detected in multiple environments. SNP markers linked to some of these QTL were converted to KASP markers, and the QTL were verified in a germplasm panel, indicating potential application in wheat breeding programs.

## Data Availability Statement

The original contributions presented in the study are included in the article and/or Supplementary Material, further inquiries can be directed to the corresponding authors.

## Author Contributions

YoZ and ZH conceived the idea. YW and XXu conducted the experiments, analyzed the data, and prepared the manuscript. YeZ, YL, ZP, YT, and DX contributed to mapping population development, phenotyping and statistical analysis. XXi and YH assisted in writing and revising the manuscript. All authors contributed to the article and approved the submitted version.

## Conflict of Interest

The authors declare that the research was conducted in the absence of any commercial or financial relationships that could be construed as a potential conflict of interest.
