# Supplementary material for: QTL Mapping for Grain Zinc and Iron Concentrations in Bread Wheat
Source: Front Nutr. 2021 Jun 9;8:680391. doi: 10.3389/fnut.2021.680391 (PMC8219861; doi:10.3389/fnut.2021.680391)
Supplement: Supplementary file 1 [file Data_Sheet_1.docx]

Fig. S1 Pleiotropic QTL effect accumulation for concentration of GZn (left) and GFe (right).


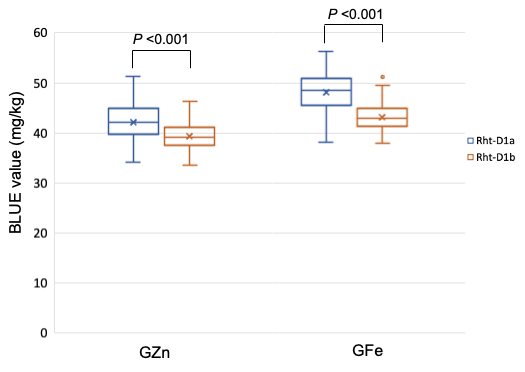


Fig. S2 Allelic effects of *Rht2* gene-specific KASP marker *K-AX-86170701* for GZn and GFe in the Jingdong 8/ Bainong AK58 RIL population; *Rht-D1a*, the allele from Jingdong 8; *Rht-D1b*, the allele from Bainong AK58.
